# Static magnetic stimulation of the primary motor cortex impairs online but not offline motor sequence learning

**DOI:** 10.1038/s41598-019-46379-2

**Published:** 2019-07-08

**Authors:** Angélina Lacroix, Léa Proulx-Bégin, Raphaël Hamel, Louis De Beaumont, Pierre-Michel Bernier, Jean-François Lepage

**Affiliations:** 1 Department of Pediatrics, Sherbrooke University, 3001-12th Ave. North, Sherbrooke, Canada; 2 Sherbrooke University Research Center, 3001-12th Ave. North, Sherbrooke, Canada; 3 Department of Psychology, Montreal University, 90 Ave. Vincent d’Indy, Montréal, Canada; 4 Department of Surgery, Faculty of Medicine, Pavillon Roger-Gaudry C.P, 6128 Montréal, Canada; 5 Faculty of Physical Activity Sciences, Sherbrooke University, 2500 de l’Université Blvd., Sherbrooke, Canada

**Keywords:** Psychophysics, Consolidation, Consolidation, Human behaviour, Human behaviour

## Abstract

Static magnetic fields (SMFs) are known to alter neural activity, but evidence of their ability to modify learning-related neuroplasticity is lacking. The present study tested the hypothesis that application of static magnetic stimulation (SMS), an SMF applied transcranially via a neodymium magnet, over the primary motor cortex (M1) would alter learning of a serial reaction time task (SRTT). Thirty-nine participants took part in two experimental sessions separated by 24 h where they had to learn the SRTT with their right hand. During the first session, two groups received SMS either over contralateral (i.e., left) or ipsilateral (i.e., right) M1 while a third group received sham stimulation. SMS was not applied during the second session. Results of the first session showed that application of SMS over contralateral M1 impaired online learning as compared to both ipsilateral and sham groups, which did not differ. Results further revealed that application of SMS did not impair offline learning or relearning. Overall, these results are in line with those obtained using other neuromodulatory techniques believed to reduce cortical excitability in the context of motor learning and suggest that the ability of SMS to alter learning-related neuroplasticity is temporally circumscribed to the duration of its application.

## Introduction

Exposure to strong static magnetic fields (SMFs) is known to bear an influence on biological systems^1,2^. At the brain level, exposure to SMF in the context of magnetic resonance imaging (MRI) has been shown to transiently modulate cortical excitability in humans^3,4^. The evidence that SMF can alter normal brain function has led to the development of a new non-invasive neuromodulatory technique called static magnetic stimulation (SMS)^5,6^. SMS relies on the application of a strong neodynium magnet positioned directly onto the scalp; this simple approach has been shown to have effects comparable to those initially observed with MRI, inducing a reduction in cortical excitability^3,6–12^, and an increase in GABA_A_-mediated intracortical inhibition^3,8,9^. While the exact mechanisms behind the inhibitory effects of SMS remain to be fully established, they could result from the induction of a rotation in cells’ membrane that causes conformational changes in transmembraneous ions channels, altering the normal passage of ions^13–15^.

Initial studies suggested that the effects of SMS on the brain were relatively short-lived (i.e., ~10 minutes)^3,6,16^, but recent transcranial magnetic stimulation (TMS) evidence shows that a 30-minute exposure to SMS over the primary motor cortex (M1) could induce enduring neurophysiological changes consistent with long-term depression (LTD)-like effects^8^. Whether these lasting LTD-like changes are strong enough to modify behaviour after the stimulation period (i.e. offline effects), or whether the behavioural effects of SMS are temporally circumscribed to the duration of its application, remains to be clarified. Similarly, it is currently unknown if the effects of SMS are spatially circumscribed to the area of the cortex directly under the magnet, or if it also impacts the functioning of other interconnected regions. Answering these questions is important to better understand the limits and capabilities of SMS, and design proper protocols for human research.

The serial reaction time task (SRTT) is a well-established task that is commonly used to assess the effect of neuromodulatory interventions on learning-related neuroplasticity^17–23^. It consists of consecutive key presses prompted by visual cues. Unbeknownst to the participant, a sequence of key presses is repeated throughout the task, which induces a gradual reduction in response time that is specific for the repeated sequence, indicative of learning. Typically, while neuromodulatory interventions applied prior to an SRTT session on the M1 contralateral to the hand performing the task influences online learning^24–27^, other evidence further suggests that similar interventions applied to the ipsilateral M1 tend to improve learning, presumably through the release of interhemispheric inhibition^20,21,28^. Moreover, low-frequency repetitive TMS and continuous theta-burst TMS applied to the contralateral M1, which are believed to induce LTD-like plasticity, appear to interfere with the consolidation processes involved in offline learning of SRTT^29–31^. The SRTT thus appears well suited to differentiate between the online and offline effects of SMS and clarify its potential distal neuromodulatory influence over the non-stimulated M1. Considering that online SMS is presumed to decrease cortical excitability^3,6,9,12^, we hypothesized that SMS applied to the contralateral M1 would impair online learning as compared to sham, while SMS over the ipsilateral M1 would improve it. In the eventuality that SMS induced lasting LTD-like effects^8^, we would expect contralateral SMS to decrease offline learning as compared to the other stimulation conditions.

## Results

### First session

**Figure 1:**
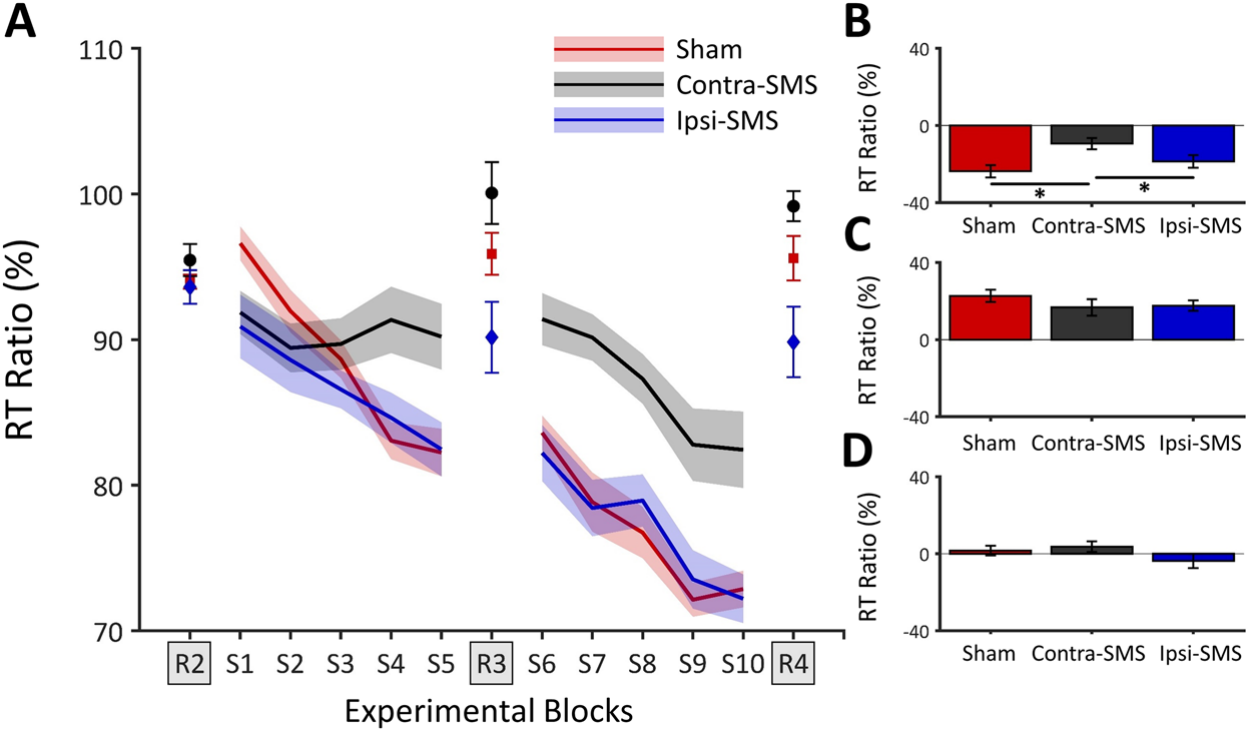
Results of the first SRTT session. (**A**) Mean RT ratio data for each group as a function of experimental block during the first session. The R1 block was used to normalize RT data into ratio. (**B**) Global learning (S10-S1) RT ratio data. The Contra-SMS group showed impaired global learning as compared to the two other groups, which did not differ. (**C**) Specific learning (R4-S10) RT ratio data. (**D**) Unspecific learning (R4-R2) RT ratio data. Error bars represent SEM. Asterisks (*) denote significant differences (*p* < 0.05).

Concerning RT data, results of the MANOVA revealed an effect of Group (*F*_(6,70)_ = 2.530, *p* = 0.028, ${n}_{p}^{2}$ = 0.178), which was broken down using the Roy-Bargmann Stepdown Procedure^32^. Concerning global learning, a univariate ANOVA revealed a main effect of Group (*F*_(2,36)_ = 5.516, *p* = 0.011, ${n}_{p}^{2}$ = 0.223), where the Contra-SMS showed lower global learning than both the Ipsi-SMS (*p* = 0.049; Cohen’s d = 0.806) and Sham groups (*p* = 0.003; Cohen’s d = 1.324). The Ipsi-SMS and Sham groups did not differ (*p* = 0.231; Cohen’s d = 0.435). Concerning unspecific learning, an ANCOVA, using the RT data from the global learning comparison as a covariate, revealed no effect of Group (*F*_(2,35)_ = 1.975, *p* = 0.154, ${n}_{p}^{2}$ = 0.101). Concerning specific learning, an ANCOVA, using the RT data from the unspecific learning comparison as a covariate, also revealed no effect of Group (*F*_(2,35)_ = 1.203, *p* = 0.313, ${n}_{p}^{2}$ = 0.064). Overall, this suggests that application of SMS selectively over contralateral M1 impaired online learning, but not global motor performance. RT data of the first session are presented in Fig. 1.

Concerning Error data, results from the MANOVAs revealed no effect of Group (*F*_(6,68)_ = 0.496, *p* = 0.809, ${n}_{p}^{2}$ = 0.042), suggesting that SMS did not influence the number of errors committed during the first session among groups.

### Offline learning

Concerning the occurrence of offline learning in RT data, results revealed that specific offline learning (S11-S10) did not occur in any groups (all *p* > 0.414; all Cohen’s dz < 0.459), but revealed that unspecific offline learning (R5-R4) occurred in all three groups (all *p* < 0.022; all Cohen’s dz > 0.756). Similar to results from Meier and Cock (2014)^33^, this suggests that offline learning of RT data occurred selectively in the unspecific comparison.

**Figure 2:**
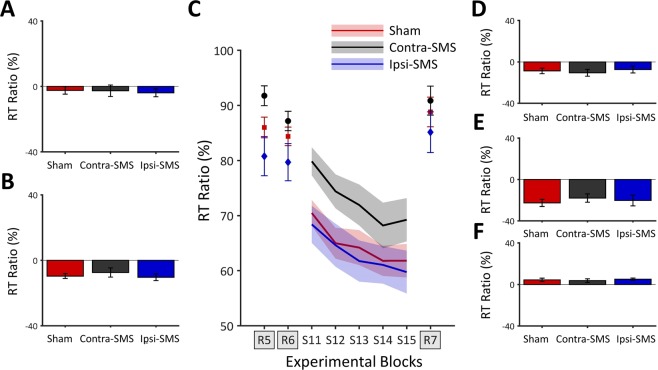
Results of the second SRTT session. (**A**) Specific offline learning (S11-S10) RT ratio data. (**B**) Unspecific offline learning (R5-R4) RT ratio data. (**C**) Mean RT ratio data for each group as a function of experimental blocks during the second session. The R1 block from the first session was used to normalize RT data of the second session into ratio. (**D**) Global learning (S15-S11) RT ratio data. (**E**) Specific learning (S15-R7) RT ratio data. (**F**) Unspecific learning (R7-R6) RT ratio data. Error bars represent SEM.

Concerning the influence of SMS on group RT data, results of the MANOVA revealed no effect of Group (*F*_(4,70)_ = 0.295, *p* = 0.880, ${n}_{p}^{2}$ = 0.017), suggesting that SMS did not influence offline learning among groups in RT data. Offline learning RT data are presented in Fig. 2.

Concerning the occurrence of offline learning in Error data, results revealed that specific (S11-S10) offline learning occurred in both the Sham (*p* = 0.015; Cohen’s dz = 0.946) and Ipsi-SMS groups (*p* = 0.009; Cohen’s dz = 1.051), but not in the Contra-SMS group (*p* = 0.239; Cohen’s dz = 0.423). Results further indicated that unspecific (R5-R4) offline learning occurred in all three groups (all *p* < 0.009; all Cohen’s dz > 0.647). These results suggest that specific and unspecific offline learning occurred in overall Error data.

Concerning the influence of SMS on group Error data, results of the MANOVA revealed no effect of Group (*F*_(4,72)_ = 1.037, *p* = 0.394, ${n}_{p}^{2}$ = 0.054), suggesting that SMS did not influence offline learning among groups in Error data.

### Second session

Concerning RT data, results of the MANOVA revealed no effect of Group (*F*_(6,66)_ = 0.812, *p* = 0.564, ${n}_{p}^{2}$ = 0.069), indicating that relearning did not differ between groups. RT data of the second session are presented in Fig. 2.

Concerning Error data, results of the MANOVA also revealed no effect of Group (*F*_(6,66)_ = 1.496, *p* = 0.193, ${n}_{p}^{2}$ = 0.120), indicating that relearning did not differ between groups.

## Discussion

This study provides evidence that SMS influences learning-related neuroplasticity in humans in a manner consistent with its documented transient inhibitory activity. On one hand, results showed that SMS applied selectively over the contralateral M1 impaired online learning without affecting offline learning or relearning measured 24 h later. On the other hand, ipsilateral SMS did not significantly facilitate, nor interfere with, online learning.

### SMS over contralateral M1 impaired online learning

The present results show that application of SMS over the contralateral M1 impaired global learning as compared to both Sham and application of SMS over the ipsilateral M1, suggesting that SMS over contralateral M1 disrupted online learning-related neuroplasticity. On one hand, these results fall in line with what is seen following putatively inhibitory rTMS, applied to the contralateral M1 prior to the learning phase^27^, and opposite to what is typically seen following excitatory neuromodulatory interventions applied to the same hemisphere^19,34,35^. Importantly, the observed effect on online learning cannot be attributed to a general slowing of motor response, as groups did not differ on RT for unspecific online learning (i.e., random sequences).

On the other hand, the apparent inhibitory nature of SMS observed here is in accordance with previous behavioural results reported in humans^16,36,37^ and animals^38^ globally showing that SMS can impair the neuronal activity mediating behavioral performance. In this light, given that SMS over M1 is known to decrease cortical excitability during and for a short period of time after its application^3,6–8,12^, SMS-induced transient disruption of M1 excitability could putatively mediate the present impairment in online learning^19,39,40^. However, since recent lines of evidence indicate that changes in M1 excitability during learning are not related to retention^41,42^, event when assessed 48 h later^43^, transient inhibition of M1 excitability during online learning may not necessarily interfere with time-dependent processes related to offline learning.

### Contralateral SMS did not influence offline learning and relearning measured 24 h later

The presents results revealed that SMS did not impair offline learning or relearning between groups measured 24 h later, suggesting that the effects of SMS were transient in nature and did not alter the neuroplastic processes within M1 that are associated with consolidation shortly after practice^31,44^. On one hand, these results contrast with what is observed with inhibitory rTMS, as perturbation of M1 before and during motor learning with rTMS can negatively impact time-dependent processes such as consolidation and offline learning^29–31,45–48^. On the other hand, these results are partly consistent with studies using anodal transcranial direct current stimulation (tDCS) over M1, a neuromodulatory intervention believed to increase cortical excitability^49^, and showing that modification of performance during online learning does not necessarily influence offline learning (for a review, see^50^). Hence, one possibility is that transient perturbations of M1 excitability occurring online do not necessarily translate into perturbations of offline learning^45^.

In stark opposition to the present findings, a recent report showed that application of SMS over contralateral M1 did not impair online learning during SRTT, but rather facilitated offline learning as compared to sham stimulation^51^. With respect to online learning, one possibility that could account for this discrepancy is the differing application duration and timing of SMS over M1 during the acquisition session of the present design as compared to Nojima and colleagues^51^. Namely, whereas SMS was applied 10 min before and during the initial SRTT session (i.e., total of ~30 min) in the present design, Nojima and colleagues (2018) applied SMS for ~10 min selectively during acquisition. Given that SMS has been shown to require at least 10 min to induce LTD-like effects on M1 excitability^6^, one likely possibility is that SMS’s impairing influence on behaviors may discerningly emerge when it has been applied for at least 10 min before learning. With respect to offline learning, although the present results argue for a null effect of SMS on offline learning, how the inhibitory influence of SMS hinges on the complex set of neural processes engaged by offline learning remains unknown. For instance, given that homeostatic and non-homeostatic interactions are known to occur between neuromodulatory interventions and motor learning sessions^52,53^, application of an inhibitory neuromodulatory intervention during learning M1 could leave unimpaired^45^, inhibit^48^ or even facilitate^51^ processes engaged by offline learning. The extent to which SMS can be used to alter time-dependent processes, such as offline learning, remains a query for future studies. Alternatively, if the short-lasting nature of SMS was to be confirmed, it would constitute a useful tool to investigate temporally-defined mechanisms involved in complex cognitive phenomena such as learning.

### Ipsilateral SMS did not impair online or offline learning

The existence of interhemispheric interactions between bilateral motor cortices is well established and appears to be mostly inhibitory in nature^54,55^. There is evidence that inhibiting one motor cortex suppresses transcallosal inhibition and leads to an increase in cortical excitability in the opposite M1 as probed with TMS^20^. In the context of SRTT, this release from inhibition induced by low-frequency rTMS has been shown to facilitate online learning in the unstimulated M1^20,21,28^. Moreover, anodal tDCS impairs online learning during SRTT when applied to the ipsilateral M1^26^, further supporting the notion that interhemispheric inhibition regulates the learning-related neuroplasticity during motor sequence learning. In the present study, SMS applied to the ipsilateral M1 did not significantly improve online learning or offline learning as one could have expected from the interhemispheric rivalry hypothesis. The precise reasons for this remain to be clarified, but one possibility is that SMS modulated different intracortical circuits than those responsible for interhemispheric inhibition (IHI). Indeed, while SMS modulates short intracortical inhibition^3,8^, these intracortical circuits have been documented to be distinct from those involved in IHI^56^, which would limit SMS capabilities to modulate IHI. Future studies combining SMS and dual coil TMS could provide additional information regarding the precise neurophysiological effect of SMS on IHI.

## Conclusion

The present results provide evidence that transcranial application of SMS over contralateral M1 impairs online learning-related neuroplasticity. The detrimental effects of SMS on motor sequence learning appeared both temporally and spatially circumscribed to the duration and brain area of application, respectively. An important step for future research would be to combine SMS with whole brain neuroimaging techniques to determine the mechanisms by which SMS influences learning-related neuroplasticity, an important step to determine SMS’ capabilities for human research.

## Materials and Methods

### Participants

Thirty-nine right-handed healthy adults with no history of neurological or psychiatric disorder participated in the study (21 females; mean age 23.28 ± 0.47 years; Mean ± SEM). Importantly, participants were carefully randomly assigned to one of the three experimental groups in order to randomize the influence of confounding biological factors on the outcome of a neurostimulation session, such as anatomy of neuronal circuits and state of M1 excitability prior to learning (for a review, see^57^). Since this work is among the first to assess the influence of SMS on learning-related neuroplasticity, the expected effect size to be achieved which could then be used to power an a priori analysis was unknown. Nonetheless, based on previous SRTT studies using other neuromodulatory interventions that had groups of four to six^31^, eight^27^ or eight to thirteen participants^29^, we made the reasonable assumption that fourteen participants per group should yield acceptable statistical power to detect a meaningful effect of SMS on SRTT. Initially, forty-two participants were collected, but three participants were excluded due to computer malfunction (n = 2) or because they did not show up for the second session (n = 1). The study was approved by the research board of the Centre intégré universitaire de santé et de services sociaux de l’Estrie – Centre hospitalier universitaire de Sherbrooke, participants gave written informed consent, and all procedures were in accordance with the 1964 Declaration of Helsinki.

### General procedure

Participants came to the laboratory for two sessions separated by a 24 h interval to perform the SRTT. Sham or active SMS (contralateral, ipsilateral) was applied during the first session only. Offline learning and relearning were assessed upon the second visit. Participants were blind to group membership, and the experimenter collecting data was unaware of the expected effects of SMS application. While double-blinding would have been preferable, we could not ensure efficient blinding of the experimenter due to the obvious interaction of the magnet with ferromagnetic objects.

### Static magnetic stimulation (SMS)

Participants were randomly assigned to one of the three following groups: sham SMS (Sham group; n = 12), contralateral SMS (Contra-SMS group; n = 13), or ipsilateral SMS (Ipsi-SMS group; n = 14). For the active SMS groups, a nickel-plated neodymium disc magnet (N52, 50 mm diameter, 50 mm thickness, axially magnetized, 139Kg pull force) was positioned over C3 or C4 sites of the 10–20 EEG system, corresponding to the left and right M1. The SMS was held in place with a custom-made helmet, resulting in approximately 180 mT reaching the cortex^58^. A non-ferromagnetic stainless steel cylinder of identical size was placed over the non-stimulated hemisphere to counterbalance the weight. For the sham group, stainless steel cylinders were placed on both hemispheres. To ensure that SMS would M1 excitability, application of SMS began 10 minutes before the onset of the SRTT^6^ and was maintained throughout the task (i.e., ~30 minutes total).

### Serial reaction time task (SRTT)

**Figure 3:**
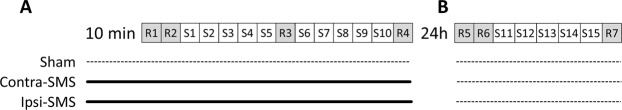
Overview of the SRTT protocol. (**A**) First session. Sham or SMS stimulation was applied 10 minutes before the onset of the SRTT protocol. SMS stimulation ceased upon first session termination. (**B**) Second session occurring 24 h later. No SMS or Sham stimulation was applied. “S” and “R” denote sequence and random blocks, respectively.

In the SRTT, a visual cue appeared alternately at one of four possible positions within a horizontal array on a screen, each position corresponding to a specific key on a keyboard. In the first session, the SRTT involved 14 blocks of 12-key sequence each, which were performed with the right hand; 10 of these blocks (sequence blocks, S1 to S10) consisted of a repeating sequence (sequence: 4-2-3-1-1-3-2-1-3-4-2-4), while the order of stimuli presentation was randomized in 4 blocks (random blocks, R1 to R4). Two of the random blocks were presented at the beginning of the task (R1, R2), and after the fifth (R3) and tenth (R4) sequence blocks (Fig. 3 for details). The second part of the task, conducted 24 h later and aiming to measure offline learning and relearning, consisted in the presentation of the same first eight blocks of the first session in the same order (i.e., hereafter referred to as R5 to R7 and S11 to S15). Reaction time (RT), corresponding to the time between visual cue onset and the key press response (i.e., hereafter referred to as RT data), and errors, corresponding to the number of incorrect responses during the task (i.e., hereafter referred to as Error data), were recorded.

### Data processing and analyses

For each participant, aberrant trials were identified within each block individually (±2.5 SD, <3% of trials) and excluded to compute representative mean RT (ms)^59^. Performance in the two sessions was normalized using the first block of the first session (R1) to compute a percentage ratio (%) for RT data. Because some participants made no error in the R1 block, Error data were not normalized.

The first analysis sought to determine whether SMS influenced global, specific or unspecific learning during the SRTT^60–62^. For that purpose, multivariate analysis of variance (MANOVAs), with Group (Sham, Contra-SMS, Ipsi-SMS) as the fixed factor were used^63^. The MANOVAs had three dependent variables, which were the RT ratio difference between S10-S1, R4-S10, and R4-R2 to assess global, specific, and unspecific learning, respectively.

To determine if offline learning occurred and differed between groups, specific learning and unspecific learning were calculated from the RT ratio difference between S11-S10 and between R5-R4, respectively. First, to determine whether specific or unspecific offline learning occurred, RT ratio difference data of each group were compared to the value 0 using one-sample t-tests, which were corrected for multiple comparisons (see below). Significant differences from the value 0 would indicate that offline learning occurred.

Second, to assess the impact of SMS on specific and unspecific offline learning among groups, the RT ratio difference data were submitted to a MANOVA using Group (Sham, Contra-SMS, Ipsi-SMS) as the fixed effect. The MANOVA had two dependent variables, which were the RT data of specific (S11-S10) and unspecific (R5-R4) offline learning. Finally, to assess the potential lingering effect of SMS on relearning during the second session, a similar MANOVA was also conducted. The MANOVA had three dependent variables, which were the RT ratio difference used to assess global learning (S15-S11), specific learning (R7-S15) and unspecific learning (R7-R6).

Moreover, to determine if the number of committed errors was influenced by the application of SMS, the same analyses were conducted on the same comparisons as with RT data but on Error data.

If a MANOVA returned a significant effect of Group, the Roy-Bargmann Stepdown Procedure was used as a follow-up analysis to determine the contribution of each dependent variable to the detected effect^32^. Briefly, the Roy-Bargmann procedure first requires the dependent variables to be ordered in descending order according to their level of theoretical importance. Then, a univariate ANOVA is conducted on the variable of highest interest. Subsequently, ANCOVAs that use the data of the immediately higher level of importance as covariates are conducted in a descending stepwise fashion to evaluate the influence of single variables on the MANOVA results^32^. In the present context, assessment of global learning was deemed to be the response variable of highest interest it was deemed to best represent learning-related performance improvements. Assessments of unspecific and specific learning were deemed to be of second and third level of theoretical importance, respectively.

For all statistical analyses, normality of distribution and homogeneity of variance were verified using Shapiro-Wilk’s^64^ and Levene’s tests (Box’s M for multivariate analyses)^65^, respectively. For post-hoc comparisons, independent t-tests or Mann-Whitney U tests were used, depending on data normality and homogeneity of variance. To control for inflated type 1 errors upon multiple comparisons^66^, the Benjamini-Hochberg procedure was used^67,68^.

## Data Availability

Data is available by contacting the corresponding author.
